# Participation in ‘big style’: first observations at the German citizens’ dialogue on future technologies

**DOI:** 10.1007/s10202-012-0119-0

**Published:** 2012-11-16

**Authors:** Michael Decker, Torsten Fleischer

**Affiliations:** Institute for Technology Assessment and Systems Analysis (ITAS), Karlsruhe Institute of Technology (KIT), Karlstrasse 11, 76133 Karlsruhe, Germany

## Abstract

In 2010, the German Federal Ministry of Education and Research started a series of citizens’ dialogues on future technologies. In the context of the German history of public participation in technology-oriented policy making, these dialogues are unique for at least two reasons: The Federal Ministry retains the responsibility for the entire process and is heavily involved in its planning, organization and communication, and the number of participants and process elements is significantly higher than in most other participative events. The paper presents insights into the political background of the citizens’ dialogues, its general concept as well as first observations from the dialogue rounds on energy and high-tech medicine. In addition, it discusses reactions of other political actors and expectations regarding legitimacy and representativeness of the dialogue results.

## Abstract (de)

In 2010 startete das deutsche Bundesministerium für Bildung und Forschung eine Serie von Bürgerdialogen zu Zukunftstechnologien. Im Kontext der deutschen Geschichte der Partizipation von Bürgern an technologiebezogenen Entscheidungen sind diese Dialoge aus wenigstens zwei Gründen einzigartig: Das Bundesministerium übernahm die Verantwortung für den Gesamtprozess und engagierte sich intensiv in dessen Planung, Organisation und Kommunikation und die Zahl von Teilnehmern und Prozesselementen war deutlich höher als bei den bislang in Deutschland durchgeführten Partizipationsverfahren. Der Beitrag stellt Einblicke in Entstehungsgeschichte, generelle Konzeption und politische Einordnung dieser Bürgerdialoge sowie erste Beobachtungen aus den Dialogrunden zu den Themen Energie und Hightech-Medizin vor. Darüber hinaus diskutiert er Reaktionen anderer politischer Akteure sowie Erwartungen hinsichtlich der Legitimität und Repräsentativität der Dialogergebnisse.

## Résumé

En 2010, le Ministère Fédéral de l’Education et de la Recherche allemand entama une série de dialogues civils sur les technologies de l’avenir. Dans le contexte de l’histoire allemande de l’implication des citoyens dans des décisions axées sur les technologies, ces dialogues sont uniques, et ce pour au moins deux raisons: Le Ministère Fédéral prit en charge la responsabilité du processus entier, en s’engageant fortement dans sa planification, organisation et communication, le nombre de participants et d’éléments du processus ayant dépassé de loin celui des processus de participation mis en œuvre jusque là. Le présent article donne un aperçu des conditions de la genèse, la conception générale et du contexte politique de ces dialogues civils et des premières observations faites pendant ces cycles de dialogue sur les enjeux de l’énergie et de la médécine high-tech. Par ailleurs, il discute les réactions d’autres acteurs politiques ainsi que les espérances quant à la légitimité et la représentativité des résultats des dialogues.

## Introduction

Political decision-making with respect to new technologies is challenged more and more by public ‘resistance’ even though there is no general technophobia in Europe.^1^ Historically rooted in the debate about nuclear power, still present with a focus on potential nuclear waste disposal sites and reactivated by the nuclear accidents in Fukushima, nowadays technologies assumed to be much less controversial get under public pressure as well. Changing an existing railway station into an underground station in Stuttgart, Germany (so-called Stuttgart 21 or S21), caused an enormous reaction by civil society at that moment when the deconstruction of the existing building of the railway station started. Interestingly, the formal planning process for S21 was correct in a sense that the usual participatory elements had taken place. There was a planning assessment procedure and it took more then a decade to get through the whole process resulting in the decision to change the railway station from a dead-end station into a pass-through underground station. Obviously, the legitimation resulting from this formal process was not perceived as ‘binding’ for a significant share of citizens in the Stuttgart region. In the case of the S21 controversy, also an ad hoc mediation process^2^ was not able to develop a solution that was considered binding by some of the groups involved. At the end of the day, a citizens’ decision was necessary to, finally, legitimize the originally developed solution of an underground railway station. Noise pollution around airports, as a third example, is also becoming more and more a matter of public opposition. Public interest groups are able to create at least regional political pressure in a way, which cannot simply be ignored by policy making. In short, the increasing complexity of both politics and polity has led to a rising dependence on formal *and* informal negotiation and deliberation to generate political legitimacy.

The underlying problem in the cases mentioned above, which are interestingly not at all restricted to ‘new technologies’ such as GMO, nanotechnology and synthetic biology, but refer to rather old technologies usually perceived as being less controversial as railways and air transport, is usually identified as being caused by insufficient integration of citizens into the political process, by inadequate communication with the people concerned by political decisions. Changes in the architecture of self-government, induced by developments that have been described as ‘monitory democracy’ (Keane 2009), defined by the rapid growth of many different kinds of extra-parliamentary, power-scrutinizing mechanisms, may also have contributed to the gradual rapprochement of institutions of representative democracy to instruments of direct democracy.

Therefore it might be not surprising that the new regional government in the State of Baden-Wuerttemberg, a coalition of the two parties BÜNDNIS 90/DIE GRÜNEN (The Greens) and the social democrats (SPD), states in their coalition treaty already at page one:^3^ ‘The time is over for top down governing. Good politics includes grass-root elements, good leadership and decision-making qualities root in the ability to listen. We see citizens’ interventions as enriching the policy making process’.^4^ Therefore, one of the four main challenges for the new government in Baden-Württemberg is ‘More citizens’ participation at all levels of decision making’.^5^

From the perspective of technology assessment in Germany, this ‘need for dialogue in the political process’ can be interpreted as a shift in thinking about participatory approaches. While in other European countries such as Denmark or Switzerland, participatory elements such as consensus conferences or ‘PubliFora’ are well established, these elements are up to now only rarely implemented in Germany. Actually, it was on the topic of nanotechnologies where a whole series of dialogues were realized by different organizations such as the German Federal Environment Agency (Umweltbundesamt, UBA) or even by industry associations or individual companies. Therefore, it was in a way surprising that the political coalition on the federal level (by the parties CDU, CSU and FDP) agreed in the coalition treaty that there should be citizens’ dialogues on future technologies. The realization of these dialogues is under the supervision of the Federal Ministry for Education and Research (BMBF) and supported by it with a solid funding. This trend is underlined by the recently finished ‘Dialogue about Germany’s future’^6^ by the Bundeskanzleramt (the Office of the Chancellor), in which the German Chancellor Angela Merkel went for town hall meetings and an online dialogue about the three main questions: ‘How do we want to live together in the future? Whereof do we want to live in the future? How do we want to learn in the future?’.^7^

Against the background of this recent trend towards a more dialogue-oriented policy-making process, we would like to introduce the citizens dialogue on future technologies of the German Federal Ministry of Education and Research from a perspective of a technology assessment institution that was (and, at the time of writing of this paper, still is) participating in the design, realization and evaluation of this process.^8^ Since the dialogue process is still ongoing, we can neither present a full description of the entire process nor is it our aim to provide a comprehensive discussion of its quality or its outcomes. This will be subject to further publications. Here, we want to describe a number of observations and present first insights and thoughts for further in-depth analysis and research, focussing on the political embedding and uptake of the dialogue which is, at least for Germany, unique because the initiating organization is a Federal ministry that not just commissioned the process to an external organization but remained in the leading role, kept its organizational and political responsibilities, and by doing this, is an active participant in the dialogue process. In the following, we describe the citizens’ dialogue (Chaps. 2 and 4) and our role in it (Chap. 3) and discuss it in Chap. 5 with respect to legitimation and the influence in the political decision-making progress.

## The citizens’ dialogue on future technologies (CDFT)

As already briefly mentioned in the introduction, the history of the German citizens’ dialogues on future technologies (‘Bürgerdialoge Zukunftstechnologien’) dates back to an agreement between the parties that currently form the parliamentary majority of the Federal Republic of Germany and therefore also the recent Federal Government. Most German Federal Governments are coalition governments, and this is also the case for the present 17th Legislative Period where a centre-right/conservative coalition of the parties CDU, CSU and FDP came into power. The political programme of coalition governments is usually negotiated immediately after electoral success and fixed in a coalition treaty. This document, agreed upon in October 2009, states:

(…) Therefore, our goal is that in Germany, the land of ideas, new technologies are not only developed but also implemented. In order to achieve this, we also need a comprehensive dialogue on future technologies with and among the citizens. We stand for a future-oriented culture of opportunities. We want to become an optimistic and technology- and innovation-friendly society again.^9^

In summer 2010, the German Federal Ministry of Education and Research (BMBF) put out a call for tender for the organization of citizens’ dialogues on future technologies. Among other goals, these dialogues should create a forum that involves citizens on a broad basis, that gives them the opportunity to inform themselves about new key technologies and research projects, that allows citizens to form an opinion in a public discourse and to advocate for the results of this opinion-forming process in discussions with policy, industry and academia. A decision on this call was made in autumn 2010: a total budget of 8 Mio € (ca. 10.5 Mio US-$) until 2013 was allocated and it was planned that four to six different fields of future technologies (‘rounds of dialogue’) should be discussed. Service contracts were issued for two lots, lot 1 being responsible for methodology, organization and evaluation while lot 2 should cover communication and public relations.

The winning consortium for lot 1 consists of four institutions: Ifok (an international consultancy company that describes itself as ‘the market leader for communication and strategy consulting on the subject of participation’), being responsible for concept development, organization, documentation and project coordination; the Institute for Technology Assessment and Systems Analysis (ITAS) at Karlsruhe Institute of Technology (KIT) (a federally funded TA research institution) with responsibilities for concept development, scientific advice and evaluation, the Department for Sociology of Technology and Environment at the University of Stuttgart (a state-funded university) leading the evaluation activities, and Zebralog (another consultancy company with expertise in ‘eParticipation’) that was subcontracted for the online dialogues.

The dialogue process was officially started in December 2010, accompanied by an opinion piece of Federal Minister Annette Schavan published in the German newspaper ‘Welt am Sonntag’ on 26 December 2010 (Schavan 2010). In this text, she explicitly formulated the goals of the dialogue process and her expectations:

Today, policymakers have to explain themselves to citizens better than before, and they must (…) offer forums where citizens can express their expectations and concerns. If you like, it’s about policy advice provided by citizens. It is about a ‘wisdom of the many.’ (…) To discuss the benefits and possible risks (of future technologies) with as many people as possible is a matter of the ‘citizens’ dialogue future technologies’, with which (…) we want to gather feedback from citizens on current innovation projects. (…) Interested citizens (will) discuss the opportunities and risks of future technologies, they can contribute the results (…) in the form of a citizens’ report in politics, science and business, and make it clear where they see opportunities and challenges (…).^10^

In the beginning, the project consortium designed a generic model for a dialogue process consisting of three phases. In the first phase, the contentual basis for the actual dialogue process was developed, using methods like focus groups and online discussions. On the basis of the outcomes of this phase, thematic frames were identified that should serve as the basis for discussion for a number of regional citizens’ conferences where they were to be debated in greater detail. Here, between 6 and 8 events with approximately 100 participants per event were planned to be held at various locations throughout Germany. This second phase also included an online component and future workshops, where appropriate. The results of these first two phases then were used as input into the final national citizens’ conference that was aiming at writing a citizens’ report. The entire process was supported by a so-called round table, that is, a board of consultants, consisting of scientists, various stakeholders (including industry associations and NGOs) and representatives of relevant Ministries. The project consortium also included an element of self-reflexivity. The entire process is evaluated by member organizations of the project consortium. The evaluation includes surveys among participants (before, immediately after, 2 months after), participant observation at various events, structured interviews with moderators, participants and members of the board of consultants. The findings of the evaluation are to be discussed immediately within the consortium in order to adjust and improve methods and outcomes already during the process.

## Excursion: why was it interesting for a TA-institution to participate in the consortium?

From a perspective of a technology assessment institution, the call for tender was interesting since the relevant aspects for participatory TA^11^ were explicitly mentioned. It was asked for the ‘wisdom of the many’ and an open discussion of chances and risks as well as opportunities and challenges of future technologies. In addition, the process provided an excellent opportunity to take cognizance of perceptions and associations, of expectations and concerns of citizens regarding societal challenges and the role of technologies to address them. We realized also tensions in the wording about the dialogues since one of the aims was to ‘become an optimistic and technology- and innovation-friendly society again’, a task which hardly might be achieved by a series of citizen dialogues, besides the fact that there is some doubt about the underlying assumption (that the German society is not technology and innovation-friendly anymore) itself.

From a methodological point of view, there was a demanding challenge. In general, a problem-oriented approach of TA (Bechmann and Frederichs 1996; Decker and Grunwald 2001) ‘follows’ the idea of designing the appropriate TA project with respect to the problem at stake (Bütschi et al. 2004, p. 16ff). This results in a combination of TA methods, which can be identified to be the most promising ones to develop potential solutions for the problem. A citizens’ dialogue is such a method to be used in a participatory TA process. It is from a methodological point of view assumed as most promising in situations in which a public debate on a technological topic is already going on, which allows for the assumption that a public opinion building process already took place (Decker and Fleischer 2010).

In the case of the CDFT, the method ‘citizen’s dialogue’ was fixed and the choice about which future technologies the dialogues would be was open. Here, the term ‘future technologies’ turns to become a methodological as well as practical challenge in itself since it can be difficult to explore how much, and what exactly, do citizens know about these future technologies. In order to overcome this methodological hurdle, the explorative approach of citizens focus groups was proposed in order to support the selection of the technologies to be debated and, after this decision was made, to gain deeper insights about the issues considered by the citizens as being relevant and the ‘if and if yes how’ citizens think and talk about the technologies at stake.

In concrete, the following three research questions were at the core for the motivation to join the ‘citizens’ dialogues’ consortium:

- to gain deeper insights into formats and methods used in ‘big size’ deliberative processes, to gather practical experience with using them, and to learn more about challenges and limitations of the respective approaches, especially with regard to the problem of aggregating the various positions voiced by the citizens in the various regionally distributed dialogue elements;
- to better understand the challenges of translating outcomes of deliberative processes into political action at the science–policy interface. Until now, most participatory exercises used an organizational model that resembled research projects. Here, funded by governments or research institutions, scientific and/or not-for-profit organizations developed a concept and organized dialogue events, analysed the outcomes and presented the documentation to its political sponsors. These constellations easily allow political bodies to criticize, downplay or even ignore the results of these exercises, especially when they contradict or defy their current political preferences or priorities. The ‘citizens’ dialogues on future technologies’ are different in this regard. It is—to our knowledge—the first case in Germany where the Federal Government retains responsibility for the entire process. The Ministry remains in the lead and is heavily involved in the planning, organization and the communication of the process. This on one side creates a much higher level of obligation on the government’s side. This involvement might also be a reason for additional negotiations between the Ministry and the consortium since the political expectations needed to meet the scientific rationality in optimizing the dialogues. Based on this, we also expected to get—as participant observers—some insights into the expectations of government officials on dialogue processes and into the reception of its results—and the problems related to that—in government institutions; and
- in the established practice of self-reflexivity in TA to test claims like that—in contrast to more expert based assessments—participative elements in technology assessment and technology governance can increase the quality and the robustness of social knowledge.

## The process so far

The influence of the Ministry as dialogue partner can clearly be seen in the following description of the process so far from our perspective. It was planned on a general level to choose future technologies as dialogue topics which are relevant and in the responsibility of the Ministry. Therefore, the first round of dialogues started in winter 2010. The Ministry decided to go for the topic ‘high-tech medicine’ as the theme for the first dialogue, not least because 2011 was declared being the ‘Year of Health Care’. There was no consultation process with the consortium on the topic on this level. The ministry even defined three subtopics: telemedicine, intensive and palliative care and neural implants. As a consequence, although communicated as ‘the first’ dialogue, it actually consisted of three loosely connected dialogue themes.

Three independent focus groups were performed in January 2011 in order to discuss people’s intuitions, associations and perceptions with regard to the respective topics, to generate additional ideas for discussions at the citizens’ conferences and the citizens’ summit, and to test and improve our input papers. In a way, the focus groups were also designed as a pretest for the large-scale empirical modules. Subsequently, the input papers for the citizens’ conferences were rewritten according to the suggestions from the focus groups participants. Venues for the citizens’ conferences were booked; members for the board of consultants were solicited, a meeting of the board was scheduled.

These plans were almost immediately put to a halt when on March 11 and the following days, three Japanese nuclear power reactors melted down. A few days later, the German federal government decided to do another U-turn in energy policy, especially with regard to nuclear energy: In late 2010, the government, after intensive lobbying from industry and using ‘meeting of CO_2_ reduction targets’ as the political justification, decided to postpone the phase-out of nuclear power—that in 2000 after years of political struggle originally was agreed on to leading to the shutdown of the last reactor somewhere between 2022 and 2024—until 2036. This decision created substantial political and public opposition. The event in Fukushima changed Germany’s Nuclear Energy policy again. The government decided to temporarily shut down a number of nuclear power plants immediately after the event, followed by a decision for a permanent closure of six nuclear power plants (the oldest, which went online before 1981) and two more that have been offline already for a few years because of technical problems some weeks later. The remaining nine plants will be shut down between now and 2022.

In the same course of events, it was decided to start a citizens’ dialogue on energy immediately and to postpone the citizens’ dialogue on high-tech medicine. Due to time pressure, also the ‘rules of the game’ were changed. Focus groups for preparations were skipped. The content of the input paper for the citizens’ conferences was mainly developed on the basis of issues and conflicts discussed at the public hearing of experts and stakeholders of an ad hoc ethics panel that was convocated by the German Chancellor to reflect on the so-called Energiewende. Additional input was provided by the representatives of the ministry. Questions regarding the general political decisions and the abrupt change of course were to be avoided, the key question was: ‘How can we shape the change?’ Eight 1-day citizens’ conferences and 22 citizens’ workshops were performed between July and September in different metropolitan areas throughout Germany. The 2-day final conference, a so-called citizens’ summit (Bürgergipfel), took place in Berlin in November 2011, where the citizens’ report was handed of to the Federal Minister Annette Schavan.

In September, the citizens’ dialogues on high-tech medicine were continued. For that topic, six citizens’ 1-day conferences in 6 different metropolitan areas throughout Germany (two per subtopic telemedicine, intensive and palliative care and neural implants) were performed. In December, the 2-day citizens’ summit on high-tech medicine was held in Berlin in one of the main parliamentary buildings. Here, again, the minister participated in day 2 of the event and accepted the final report developed by the citizens. The entire documentation of the two dialogue processes, including the written documentation of the citizens’ conferences and the citizens’ summits, can be found on the Internet.^12^

## Discussion

A more in-depth comparison between the different ‘rounds of dialogues’ and its outcomes will have to be reserved to a future publication, once the CDFT process is finished. In this text, we would like to present first observations and a number of research questions, focusing on the potential impacts of the CDFT and lessons for the application of deliberative methods in technology assessment.

The starting point for these considerations is that technological developments are neither deterministic nor linear, but that they are socially shaped (Bijker and Law 1992; Williams and Edge 1996). Various actors, their technological and societal agendas as well as structural and political conditions (rules, conventions, mechanisms, processes) are dynamic elements in this process of mutual guiding and shaping technological configurations, also referred to as technology governance. In other words, the future direction of technological developments is open to the exercise of human agency and deliberate choice.

Technology assessment can be described as problem-oriented research into the—actual or hypothetical—impacts and consequences of the application of (new) technologies which is required to investigate a combination of ecological, social and economic conditions, to identify relevant normative frameworks such as sustainable development, and to prepare its results ‘ready for decision making’ for policy makers (Bechmann et al. 2007) as well as for informing society at large. Historically, TA was intended to serve as an ‘early-warning system’, an institutionalized way to initiate a science-based form of analysing and evaluating actual and potential societal impacts of technological innovations and advising policy making in dealing with the identified challenges and impacts. Basically, a technocratic exercise in the hands of experts in its first years, TA has regularly faced fierce criticism, both as theoretical and as political concept. In response to that, various new approaches to and forms of TA^13^ have been developed and tested that intended to address some of the criticism (Grunwald 2002).

Participatory technology assessment (pTA)—one of these new TA concepts—emerged almost in parallel with increasing demands for extended (more, better) citizens’ participation in political processes that can be observed since the 1990s. Although various models and procedures for pTA have been developed and tested within both national and European projects,^14^ and scholarly interest in the development and assessment of pTA has increased significantly, the state of pTA—especially when aiming at the inclusion of members of the general public—can still be described as experimental. Neither are pTA procedures well established in most countries (Switzerland and Denmark are often mentioned as the sole exceptions), nor is there any broadly shared agreement on good practices of pTA, nor is it so far sufficiently theoretized.^15^

One of the reasons for that might be that there are different—and competing—conceptualizations of participation. Like other social actors, also scholars and practitioners of TA act and reflect from a particular participation rationale also when doing research, developing or adapting theories^16^ and designing interventions. A look into the pTA literature shows a large variety, a pluralism of these participation rationales. Some scientists argue that deliberation improves the quality of information that can be used in the policy process. In other words, deliberation enhances the epistemic foundations of policy making.^17^ (And this basically also appears to be the dominant political rationale behind the BMBF initiative on CDFT). Others assume that deliberative outcomes are qualitatively different rather than simply epistemically better. Deliberation in that case is seen to promote values such as justice, autonomy, legitimacy and equality. This pluralism also results in different assumptions or claims about the role of pTA in the policy-making process, considering it, for example, as a means of participatory governance (Aichholzer et al. 2010) or an instrument for the democratization of expertise (Abels and Bora 2004).

Some typologies of deliberation rationales have been proposed. Very often referred to is a typology first described by Fiorino (1989) that differentiates between normative (participation is just the right thing to do), substantive (aims at generally achieving ‘substantially’ better ends) and instrumental (aim to achieve a particular predefined end) rationales. While Fiorino’s approach has been developed from an analysis of the scholarly literature, Anna Wesselink and colleagues have discussed (Wesselink et al. 2011) practitioner’s perspectives on participation and found a fourth rationale what they describe as legalistic (participation is only organized to meet formal requirements). Most authors confess that this systematic does not provide for a clear analytical distinction but that there in practice may be overlaps between different rationales.

Against the background of our expertise with applying deliberative methods in technology assessment and policy advice, we would like to encourage further scientific research on the underlying motives and rationales for using them in different contexts. In our opinion, the application of these methods within TA always has an instrumental dimension. It can be described as a reaction on a situation that includes—or is the consequence of—(perceived) policy deficits or policy failures (Abels 2009). Therefore, deliberative elements always include or refer to the promise that their application can or will improve this situation. In addition, both the deficit analysis and the justification for the use of deliberative methods have a normative dimension, they have normative foundations.

The seemingly dominant role of instrumental and legalistic rationales are relatively neglected in academic commentary or, when discussed, more of than not, flatly denounced—apparently because they conflict with the normative rationale that seems to be prevalent in scholarship on deliberative and participatory decision-making. In other cases, for instance in some fields of problem-oriented research like in sustainability studies or in climate science where it is regularly argued by some scholars^18^ that extensive participation would be required to bring about desired changes, instrumental rationales are presented (or disguised) as normative ones.

New research into participatory technology assessment should take account of this pluralism of participation rationales and also of the policy context in which participation takes place (Renn 2001). A deeper understanding of these justifications and their contexts may also foster more reflexivity among scientists who propose and use deliberative elements, especially that simply implementing participation is not going to solve larger problems of political priories and preferences.

Just to mention a theoretical reference point, concepts introduced by Andy Stirling might be helpful to structure this research problem. Stirling proposes a distinction between ‘commitment’ and ‘appraisal’ in technology governance, two processes that are different in its roles but at the same time parallel, interlinked and mutually co-constituting: ‘(…) appraisal is about *informing*, and commitment is about *forming* tangible social choices in the governance of science and technology’ (Stirling 2008). Here, appraisal comprises essentially ‘epistemic’ processes, ‘ways of understanding’ the socio-technical system, where knowledge is produced and filled with meaning, where understandings are developed and social learning occurs (Smith and Stirling 2007). This idea can—with some loss of complexity—be translated into the language of the practice of technology assessment. Stirling’s ‘appraisal’ corresponds to a core element of a stylized TA project approach: Situation appreciation (Bütschi et al. 2004, p. 19). Both can fulfil different tasks in a certain societal context: they can be about ‘closing down’ or ‘opening up’ (Stirling 2008) processes of problem framing and choice of science and technology policy options.

Deliberative exercises can play a valuable role especially in ‘opening up’ approaches. With the help of the participants, questions alternative to those discussed by institutionalized stakeholders can be asked, neglected issues and marginalized perspectives can be (re-)discovered, ignored or overseen uncertainties can be integrated, different possibilities for governance approaches can be examined and new options developed. When revealed to wider technology governance and science technology policy discourses, they may contribute to more robust social knowledge and higher levels of transparency and accountability. The idea of using lay knowledge to broaden and enrich the societal debate about new technologies and technology-related policies was also one of the constitutive ideas of the citizens’ dialogue on future technologies.

A comprehensive discussion of the entire dialogue process on the basis of two cases is not very sufficient. However, on a general level, one can already identify differences between the two dialogues which can be explained by the topics to be discussed. In the dialogue on ‘Changing the Energy System’, the citizens’ comments referred to the society as a collective. It was about the measures to take as a society in order to transform the energy system and designing a future electricity infrastructure without nuclear power. The wording was about ‘we should go for a decentralized system’, and the federal political institutions should ‘realize a strong governing in the transformation process’. For the dialogue on high-tech medicine, the statements were strongly related to an individual’s point of view. Individuals who have health problems seek solutions within a health care system that exists within a society and is shaped by it. Therefore, a number of topics explicitly addressing this constellation were mentioned, such as informed consent for patients’ decision-making and aspects of distributional justice, especially the expectation to avoid a ‘two (or more) classes’ medicine.

Although it is too early for an elaborate discussion of the impact of the dialogues, two results of the dialogue on energy should be briefly mentioned. So far, the responsibilities for energy policy were distributed among various German Federal ministries. In the dialogue, citizens have proposed the creation of an energy ministry. This idea that also was supported by experts for years but so far proved to be rather unpopular among policy makers now gained new political momentum. The minister herself also introduced the results of the citizens’ report in a meeting of the so-called research union industry–science, which advises the research ministry on innovation policy. This group appreciated the report and indicated interest to integrate some of the results in the elaboration of its recommendations and proposals for future political action.

### Is there already sufficient empirical evidence for the claimed advantage of participatory/deliberative elements in policy making?

The discussions within the coordinating group for the CDFT as well as discussions between the representatives of the Ministry for Education and Research and the scientists within the coordinating consortium point towards another potential line for further research in public participation and deliberation. As previously described, participative or deliberative elements are seen as instrumental by many policy makers. Their use is proposed as a reaction on a situation that is perceived as being in deficit. At the same time, deliberation is still an unconventional, broadly unknown method in practical policy making. Therefore, scientists interacting with policy makers and civil servants in the design of participative or deliberative elements are constantly asked to provide justifications that the deliberative approach is better than the established ways of policy making. In these interactions, the ‘gold standard’ for acceptance of these deliberative approaches is ‘empirical evidence’. In general, we found that the empirical literature on participation in technology-oriented policies and decision-making was very project specific,^19^ presenting observations and outcomes of isolated deliberative exercises, and provided divergent findings with very limited potential for comparative analysis. It remains unclear whether these differences depend on different national policy-making cultures, design or method flaws or failures, the subject (technology) under discussion, or other factors. It is our hope that the ‘big style’ of CTFD, where a number of technologies and issues are discussed in comparative formats and within a single national culture, can on the long run provide some deeper insights.

In addition, we think that it would be helpful to work towards a form of theory of deliberation that can be characterized as an empirically saturated mid-range theory with a potential for mid-level generalizations. This form of theory would have to be located between an idealized, purely normative and a pragmatic, largely descriptive understanding of deliberation by combining, but not mixing, normativity and facticity (Habermas 1998). It should take empirical diagnoses into account and integrate them—perhaps even constitutively—into their theory design. At the same time, the theory should remain open to normative aspects that are to be made explicit—again based on empirically saturated diagnoses. Such a programme could help to reconnect the two currently seemingly disjointed research strands—empirical analysis of deliberative processes with the intention of the review and further development of the theory—and theories development itself (for a broader discussion see, e.g., Mutz 2008 and Thompson 2008). And this of course has also an instrumental dimension—it could improve the conditions for the integration of participative and deliberative elements into existing and future policy-making structures.

### The citizens’ dialogue in the context of democratic decision-making

After these questions for further research on dialogue resp. deliberative methods, we would like to come back to the issue of the democratic relevance of the CDFT. One of the first political reactions on the announcement of the CDFT occurred in the German parliament, the Bundestag. Some parliamentarians were surprised about this process—although they could have known about it since all documents related to it were openly accessible. The biggest parliamentary opposition party (the social democrats) started a formal process.

The Bundestag has a series of instruments and measures at his disposal to scrutinize the work of the government. This means, above all, that parliamentarians are able to inform themselves about the work and intentions of the government and put critical questions. The rules of procedures of the German parliament allow all parliamentarians to formally submit questions on important policy issues and government positions which are known as ‘major and minor interpellation’. In March 2011, the social democrats submitted a minor interpellation (Bundestag 2011) that consists of 66 written questions, asking, among others, about the formal role of the CTFD: ‘How does the increasing use of Citizens’ Dialogues by the Federal Ministry of Education and Research relate to the fundamental understanding of our parliamentary democracy and the role of the members of the German parliament as the democratically elected representatives of the people?’^20^

This interpellation was answered in April. The government declared that ‘citizens’ dialogues, as now for the first time planned to a greater extent by the BMBF, are an instrument of participation of and policy advice by citizens. They cannot—and ought not—substitute political decisions by the Parliament.’^21^ This answer formally clarifies the intentions of the CDFT and the role of the parliament. CDFT are explicitly seen as a tool of the executive branch of government, the constitutional rights of the parliament will remain unaffected. In other words, CDFT can be considered as deliberative supplements to the procedures and institutions of the current German model of representative democracy, not as substitutes.

### Representativeness and legitimacy

Two topics that are closely linked with the implementation of deliberative elements in policy making in general and science and technology policy in particular are political representation and legitimacy. There is a long debate on these issues in theory of deliberative democracy (see, e.g., Manin 1987; Dryzek 2001; Fung 2003; Parkinson 2003; Urbinati and Warren 2008) and in the academic discussions about participatory technology assessment (see above). What is different from most other German pTA case studies is that in the CDFT process a) a significantly higher number of people are involved in the deliberation processes (about 1,000) and b) that the Federal government itself (represented by its Ministry for Education and Research) acted as immediate dialogue partner and the leader of the process.

The notion of representativeness is of high importance both for the Ministry and for the participants of the CDFT process. One indicator for that observation is the process design itself. Already in the early project phase, it was agreed upon a process that included two key elements in its design: A number of regional citizens’ conferences (usually between six and eight), geographically distributed over Germany with about 100 ‘representatively selected’ participants per conference—and a citizens’ summit consisting of ‘representatives’ of the various regional conferences that develops the final report for the respective round of dialogue. ‘Representatively selected’ here means the generation of statistical representation, a situation in which the composition of the participants of the deliberative elements should mirror the wider population, the German ‘socio demographic average’, considering gender, age, level of education and ‘ethnic background’.^22^ Although such a modus operandi has been criticized by theorists of deliberative democracy since ‘missing from such selection processes is the legitimating bonds of authorisation and accountability between participants and non-participants’ (Parkinson 2003), it is the common procedure for most deliberative events, mainly for pragmatic reasons, because the election of representatives from the general public—which would some consider as being more legitimate—would generate substantial expenditures.

Another point that should be reflected upon is the advantages and disadvantages of the statistical representation for the content and the quality of the dialogue. Two distinctions can be made here. The first one is linked to the question: ‘What should be represented?’ While most practitioners argue that (relevant) persons or groups should be represented, Dryzek and Niemeyer (2008) advocate the representation of discourses, ‘a set of categories and concepts embodying specific assumptions, judgments, contentions, dispositions, and capabilities’. This is an idea that *prima facie* also could have potential for deliberating the role of future technologies in society but needs further elaboration for its practical implementation and also some testing in protected spaces before being applied to deliberative exercises.

The other question to be discussed is whether different groups should be represented proportionally (like in the design chosen for CDFT) or equally. In the ideal deliberative democracy, all persons and groups should be given equal opportunity to participate and contribute to deliberation. For the organization of deliberative events which are for practical reasons limited in size and scope, this creates the challenge to form a panel with equal representation of the relevant differences. In order to be able to do so, one would have to identify all relevant positions and the persons and groups representing them in advance of the actual event, a task that itself only can be fulfilled within a deliberative exercise. This creates a kind of a start-up problem (Anfangsproblem) for the organizers which is usually avoided by choosing proportionality. On the other hand, one should be aware of the fact that—depending on the topic to be negotiated—also within larger panels like in CDFT, some positions will remain unrepresented in the deliberations. For many practitioners, this appears to be acceptable, especially for formats whose aim is information-gathering (or ‘opening up’ the social appraisal of technologies, as explicated above) rather than decision-making. But also these exercises contain elements of decision-making, for example, when deciding on topics to be deliberated or when developing recommendations, where majority votes can—and in the case of the CDFT citizens’ conferences have—influence the actual outcomes.

Beyond that, further challenges result from the scale problem of deliberative approaches. Generally speaking, the number of citizens usually invited for participatory and deliberative processes depends on the methodological approach. For an in-depth discussion with extensive argumentation, a number of persons between around ten and a maximum number of 20 is considered as being optimal, for example. In this case, controversies can be discussed until an agreement is reached and ‘ideal’ deliberation seems to be possible. If voting on existing statements after discussing the topics in the plenary is sufficient for the actual contexts, groups of 30 to a maximum of 80 Persons are manageable. Besides the interplay between group size and deliberative quality, the economy of deliberative approaches is another limiting factor. Obviously, participation in ‘big style’ needs a large amount of resources on the side of the organizers as well. A citizens’ dialogue with ten tables à ten persons needs a large number of moderators and note takers at the tables as well as background staff in order to get the different results combined into an integrated final report.

The design of the CDFT created a situation where—other than in most other^23^ deliberative events—in this special case up to or even more than 1,000 citizens per topic are involved in a dialogue process. In other words, the number of participants in the dialogues is one or two orders of magnitudes higher than in many other participatory events and lies in the range that is usually considered as being representative in quantitative surveys in Germany.

This might have influenced both the expectations on and the perceptions of the outcomes of the dialogue process. During the first two rounds, we have observed a number of hints that support the relevance of this point. It was not only the ministry that was pushing the organizers to guarantee a ‘representative’ composition of participants in the various events. Also a number of dialogue participants approached members of the organizing teams and shared their impressions that they perceive the panels as being not representative for the society at large. They especially mentioned that they felt that younger people or people with a migration background were under-represented. It seems that there is something like an ‘expected representativeness’ of the panels among organizers and participants in a dialogue process. To better understand this phenomenon is a subject for further research in the next round of dialogues and in other deliberative exercises. It would be of interest to deeper investigate these expectations and how they relate to the acceptance and the legitimacy of deliberative processes and its outcomes, especially when taking account of Parkinson’s (2003) summary of the academic debates about legitimacy in deliberative democracy: that legitimacy is a regulatory ideal, not a fixed point on a scale, and that ‘genuine legitimacy is built over time by the discursive, critical examination of institutions and their actions such that ‘people actually consider institutional arrangements to be in their interest’ and such that ‘institutional arrangements actually are in everyone’s interest”’ (p. 184).

At the end of our discussion, we want to further reflect on these expectations with respect to representation among participants and the research ministry by performing a thought experiment. As mentioned previously, the overall number of participants in the CDFT was close to 1,000 persons per ‘round of dialogue’. Imagine that the organizers would have been able to design this sample in a way that it mirrors the representative ‘average’ of Germany. The basic organizational module in all citizens’ conferences and citizens’ summits was table groups of ten persons discussing topics they decided upon, which allowed for detailed argumentation and extensive deliberation. Imagine further that the organizers were successful in compiling the different results from the table discussions into the respective reports, in transferring the results of the regional citizens’ conferences to the citizens’ summit in Berlin, and finally, that the citizens’ summit itself succeeds in developing a consensual report which was handed over to the minister. In such a case, the results of the citizens’ dialogues could be perceived as being representative or—to push things just a little bit further—could be communicated by interested groups as valid representative qualitative results. The outcomes could be considered as ‘representative expectations’ on future actions of the Federal government. Given the high legitimacy that results from representative exercises usually have in German society, this would raise the pressure on the government to actually implement the recommendations of dialogue process and not just to consider it as ‘another form of advice’, and at the same time, it would certainly create tensions with the established ways of political decision-making, including the parliament (see Sect. 5.3).

## Footnotes

1 Sixty-six per cent of the EU27 average agree to the statement “Science and technology make our lives healthier, easier and more comfortable”. Even though this represents less agreement than in the year 2005 (78 % EU25 average), it is still far away from technophobia (Euro 2010, p. 32).

2 The mediation was conceptualized and moderated by Dr. Heiner Geißler and published in Geißler (2010).

3 The coalition contract can be found at http://www.gruene-bw.de/fileadmin/gruenebw/dateien/Koalitionsvertrag-web.pdf.

4 Original: “Die Zeit des Durchregierens von oben ist zu Ende. Gute Politik wächst von unten, echte Führungsstärke entspringt der Bereitschaft zuzuhören. Für uns ist die Einmischung der Bürgerinnen und Bürger eine Bereicherung. […] Wir wollen Baden-Württemberg zum Musterland demokratischer Beteiligung machen”.

5 Original: “Mehr Bürgerbeteiligung auf allen Entscheidungsebenen”.

6 Original: “Dialog über Deutschlands Zukunft”.

7 Original: “1. Wie wollen wir in Zukunft—in fünf bis zehn Jahren—zusammenleben? 2. Wovon wollen wir in Zukunft leben? 3. Wie wollen wir in Zukunft lernen?”

8 The authors are well aware of the methodological problems that may emerge from this double role as actors and participant observers. However, the close collaboration with the ministry would not allow for an additional external observer. Therefore, the “internal observer’s” perspective is the only one available.

9 Original: “(…) Deshalb geht es uns darum, dass in Deutschland, dem Land der Ideen, neue Technologien nicht nur entwickelt, sondern auch angewandt werden. Dazu brauchen wir auch einen umfassenden Dialog über Zukunftstechnologien mit und unter den Bürgerinnen und Bürgern. Wir stehen für eine zukunftsorientierte Kultur der Chancen. Wir wollen wieder eine optimistische und technik- und innovationsfreundliche Gesellschaft werden”. The full text of the coalition treaty (in German) can be found at http://www.cdu.de/doc/pdfc/091026-koalitionsvertrag-cducsu-fdp.pdf.

10 Original: “Politik muss sich heute gegenüber den Bürgerinnen und Bürgern besser als bisher erklären und sie muss (…) Foren anbieten, in denen Bürger ihre Erwartungen und Bedenken artikulieren können. Wenn man so will, geht es um Politikberatung durch Bürgerinnen und Bürger. Es geht um eine "Weisheit der vielen”. (…) “Nutzen und mögliche Risiken (von Zukunftstechnologien) mit möglichst vielen Menschen zu debattieren, ist ein Anliegen des “Bürgerdialogs Zukunftstechnologien”, mit dem wir (…) Rückmeldungen der Bürgerinnen und Bürger zu aktuellen Innovationsvorhaben einholen wollen. (…) Interessierte (werden) die Chancen und Risiken von Zukunftstechnologien diskutieren und die Ergebnisse (…) in Form eines Bürger-Reports in Politik, Wissenschaft und Wirtschaft einbringen können und so verdeutlichen, wo sie Chancen und Herausforderungen (…) sehen”.

11 See for example Skorupinski and Ott (2000) or Grunwald (2002), 127ff.

12 http://www.buergerdialog-bmbf.de. Please note that all reports are available in German language only.

13 E.g. Constructive TA, Real-Time TA, see Grunwald (2002) for an overview.

14 For example EuropTA project (Klüver et al. 2002; Joss and Bellucci 2002).

15 Cf. for example van Oudheusden (2011) and the comment on it by Guston (2011).

16 Theoretical “classics” of deliberation theory that can be considered as being inspirational for the justification of pTA approaches are, e.g., Habermas (1981), Renn and Webler (1996), Rawls (1997) and Dryzek (2000).

17 In the context of mode 2 knowledge production (Gibbons et al. 1994, p. 7) or transdisciplinary research with respect to environmental policy (Häberli and Grossenbacher-Mansuy 1998, p. 200).

18 For example Stauffacher et al. (2008).

19 For example, the Flamish Nanodialogue (van Oudheusden 2011; Guston 2011), Meeting of Minds (Abels and Mölders 2007), WWViews on Global Warmiung (Knapp and Hauser 2011).

20 Frage 65: Wie verhält sich die zunehmende Nutzung von Bürgerdialogen durch das BMBF zum grundlegenden Verständnis unserer parlamentarischen Demokratie und der Rolle der Mitglieder des Deutschen Bundestages als demokratisch gewählte Vertreter des Volkes?

21 Antwort: Bürgerdialoge, wie sie das Bundesministerium für Bildung und Forschung nun erstmals in größerem Umfang plant, sind ein Instrument der Partizipation und der Politikberatung durch Bürgerinnen und Bürger. Sie können und sollen die politische Entscheidung durch das Parlament nicht ersetzen.

22 One should note that the recruitment by random telephone calls, which included an element of self-selection, led to the well-known effect that the panel in practice showed a bias towards older, male participants with higher education.

23 There were few events with a similar size, like “Meeting of Minds” or “WWViews on Global Warming”, but, in contrast to the CDFT case, they were exercises on a multinational level. http://www.meetingmindseurope.org, www.wwviews.org.
